# Antidepressant Shugan Jieyu Capsule Alters Gut Microbiota and Intestinal Microbiome Function in Rats With Chronic Unpredictable Mild Stress -Induced Depression

**DOI:** 10.3389/fphar.2022.828595

**Published:** 2022-06-13

**Authors:** Jingxuan Tan, Xixuan Li, Ying Zhu, Mitchell A. Sullivan, Bin Deng, Xuejia Zhai, Yongning Lu

**Affiliations:** ^1^ Department of Pharmacy, Union Hospital, Tongji Medical College, Huazhong University of Science and Technology, Wuhan, China; ^2^ Glycation and Diabetes Group, Mater Research Institute- University of Queensland, The Translational Research Institute, Brisbane, QLD, Australia; ^3^ Hubei Clinical Research Centre of Precision Drug Use for Major Diseases, Wuhan, China

**Keywords:** shugan jieyu capsule, chronic unpredictable mild stress, depression, intestinal flora, 16S rDNA amplicon sequencing

## Abstract

Shugan Jieyu Capsule (SG) has been widely used in China to treat mild to moderate depression. *Hypericum perforatum* L. (St John’s Wort, SJW) is the main ingredient of SG and has been used as herbal medicine to treat depression in western countries. However, it is known that SJW has low bioavailability and does not easily get through the blood-brain barrier. Therefore, how SG plays an antidepressant effect in the central nervous system (CNS) remains an urgent problem to be solved. Mounting research has described the relationship between antidepressants and intestinal microbiota to illuminate antidepressive mechanisms in the CNS. We aimed to investigate the effects of therapy with SG on the function of gut microbiota and intestinal microbiota in rats with chronic unpredictable mild stress (CUMS)-induced depression. The psychophysiological state and the hypothalamic-pituitary-adrenal axis function of rats are evaluated through behavioral experiments, corticosterone levels, serotonin levels, and adrenal index measurements. 16S rDNA amplicon sequencing is used to test the changes in gut microbiota and make functional predictions of genes. With treatment of SG, the depression-like behaviors of CUMS-induced rats were reversed; the corticosterone levels and the adrenal index decreased significantly; the level of serotonin increased significantly; and the alpha and beta diversity analysis of microbiota showed an increase in the richness and uniformity of the flora were increased. SG regulated the relative abundance of *Actinobacteria,* Erysipelotrichaceae, Bifidobacteriaceae, Atopobiaceae, *Dubosiella*, and *Bifidobacterium*; Linear discriminant analysis effect size analysis demonstrated that Lactobacillaceae (family level), *Lactobacillus* (genus level), *Lactobacillales* (order level), *Bacilli* (class level), and *Lactobacillus-reuteri* (species level) were biomarkers in the SG group samples, and also likely to modulate metabolic pathways, such as those involved in carbohydrate metabolism, amino acid metabolism, and signal transduction. These data clearly illustrated the effect of SG on gut microbiome, thus laying the foundation for uncovering more insights on the therapeutic function of the traditional Chinese antidepressants. The potential of SG on mechanisms of antidepression to alter gut microbiota and intestinal microbiome function exposed to CUMS can be explored.

## Introduction

Depression, a highly prevalent mental illness, is estimated to affect nearly 4.4% of the world’s population (Young et al., 2021). The incidence and death rates of depression have increased in recent years, becoming an increasingly heavy burden on individuals and society, especially during the COVID-19 pandemic (Malhi and Mann, 2018; Salari et al., 2020). Predominant theories explaining the underlying pathophysiology of depression include but are not limited to: activation of the inflammatory response system; deficiency in monoamine neurotransmitters; hypothalamic-pituitary-adrenal (HPA) axis dysfunction; and low expression of brain-derived neurotrophic factor (Zhang Y. et al., 2021). Commonly, adverse reactions of antidepressants are on the digestive system and nervous system, which can result in poor patient compliance and a high recurrence rate (Kostev et al., 2014; Li et al., 2019). Therefore, there is an urgent need to effectively address depression with treatments that have fewer side effects.

Recently, patients and physicians have become increasingly interested in natural products because traditional herbs provide a prospective alternative in the treatment of depression. Shugan Jieyu Capsule (SG), a Chinese herbal medicine mainly composed of *Eleutherococcus senticosus* (Rupr. and Maxim.) Maxim. and *Hypericum perforatum* L, is a novel Chinese medicine approved by SFDA to treat mild to moderate depression (Yao et al., 2020). It is now used as a proprietary Chinese medicine in the treatment of depression clinically and has been reported to exhibit a significant antidepressant effect (Fan et al., 2021). *Eleutherococcus senticosus* (Rupr. and Maxim.) Maxim. has been reported to be anti-fatigue and anti-stress and has been described to regulate cellular immunity and humoral immunity (Shaaban et al., 2019). *Hypericum perforatum* L, also known as St. John’s Wort (SJW), is the main antidepressant medicinal component of SG (Galeotti, 2017). In 1984, Germany listed SJW as an antidepressant in its pharmacopeia. Research on the antidepressant effects of SG and SJW has mainly focused on the hypothesis that they address monoamine neurotransmitter deficiency (Fu and Liu, 2016; Zirak et al., 2019). However, it has been reported that SJW has low bioavailability and does not easily get through the blood-brain barrier (Guo et al., 2012). It is therefore worthwhile to search for mechanisms by which SG and SJW exert an anti-depressant effect via the central nervous system (CNS) instead of getting through the blood-brain barrier.

Mounting evidence has indicated that there is communication between intestinal flora and the CNS via a complex multi-organ bidirectional signaling procedure (Cryan et al., 2019; Lai et al., 2021). Studies have revealed that the gut microbiota is closely related to the pathogenesis of depression, but the underlying mechanism is unknown. For example, researchers analyzed the fecal samples of 46 patients with depression and 30 controls and found that the intestinal flora composition significantly changed in patients with severe depression (Jiang et al., 2015). Furthermore, psychotropic drugs’ effects on the function and composition of gut microbiota have also received attention recently. For example, administration of the antidepressants fluoxetine and amitriptyline has been reported to induce alterations in the gut microbiota of a rat model of depression, and various antidepressants have demonstrated different effects on intestinal flora (Zhang W. et al., 2021). Exploring whether SG influences the intestinal microbiome and whether it plays a role in the CNS may help explain the reported benefits of SG in the treatment of depression.

Thus, this study aimed to evaluate the effects of SG on the intestinal flora and the level of depression in rats exposed to chronic unpredictable mild stress (CUMS). To assess the levels of depression, two behavioral tests were conducted: the open field test (OFT) and the sucrose preference test (SPT). We detected the levels of plasma corticosterone and measured the adrenal index to evaluate whether SG administration affects the HPA axis. Plasma serotonin (5-HT) was investigated to evaluate whether SG could regulate the level of neurotransmitters. We also determined the effect of SG treatment on bacterial community structure and distribution and made gene functional predictions based on 16S rDNA amplicon sequencing. Our data help provide a new perspective on the mechanisms of SG in the treatment of depression. The population of intestinal microbe and the engagement of specific bacterial species may be involved in predicting the occurrence, development, and prognosis of depression in the future.

## Materials and Methods

### Animal Model and Experimental Timeline

**FIGURE 1:**
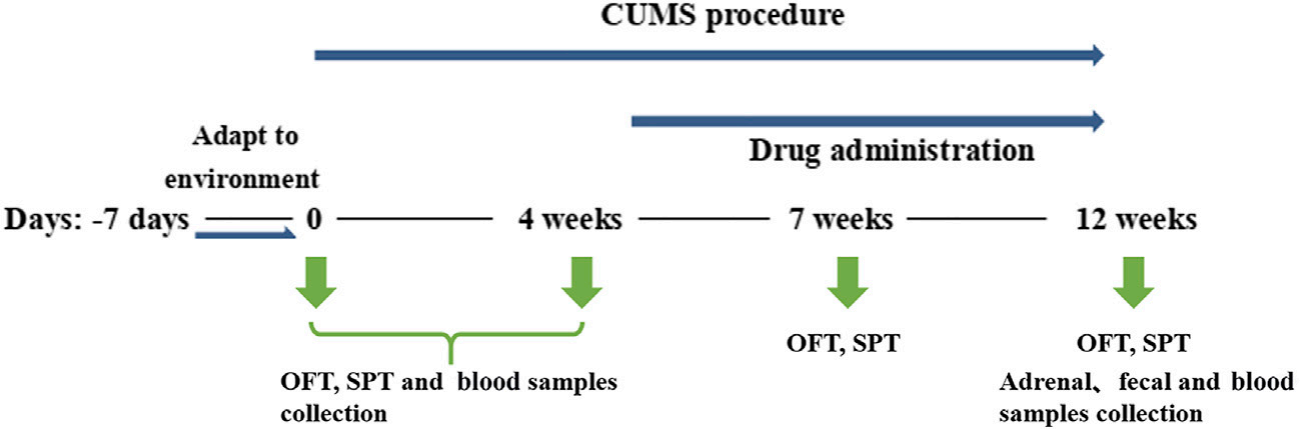
The experimental timeline. Before the CUMS procedure and by the end of the fourth week, SPT and OFT were performed and blood samples were collected. By the end of the seventh week, SPT and OFT were performed in each group of rats. By the end of the 12th week, SPT and OFT were performed and the blood, fecal and adrenal samples were collected.

The animal protocol for this study was approved by the Institutional Animal Care and Use Committee at Tongji Medical College, Huazhong University of Science and Technology (Permit Number [2021]IEC (347)). Specific-pathogen-free adult Sprague-Dawley male rats weighing 180–220 g were from the Laboratory Animal Center, Huazhong University of Science and Technology (Wuhan, China). Overall, the animal study lasted for 13 weeks as shown in Figure 1. Eighty rats were housed for 1 week in a controlled environment (12 h light/dark cycle, 23 ± 1°C, and 60 ± 5% relative humidity), with ad libitum access to food and water. After 7 days’ acclimatization, OFT was performed to ensure the homogeneity of rats. The remaining 75 rats were randomly divided into five groups, the Healthy Control group (HC group; n = 15), Model group (CUMS group; n = 15), CUMS + fluoxetine group (Flu group, 20 mg kg^−1^, q. d.; n = 15), CUMS + *Hypericum perforatum* L. Group (SJW group, 204 mg kg^−1^, q. d.; n = 15), and CUMS + Shugan Jieyu Capsule Group (SG group, 150 mg kg^−1^, q. d.; n = 15). The dosage of fluoxetine, SJW extracts, and SG was the therapeutic dosage recommended for clinical trials (rat dosage = human equivalent dosage ×6.2) (Group, 2002; Yao et al., 2020). Flu and SJW groups were used respectively as positive controls for a chemical drug and a traditional Chinese medicine. Except for the HC group, the rats continued to be given modeling stimulation until the end of the study. The SPT and OFT were performed in each group of rats to evaluate their depressive states, and blood samples were collected to evaluate their levels of corticosterone and 5-HT. Adrenal samples were collected to measure the adrenal index, helping evaluate the HPA axis system. The antidepressant groups were respectively administered with Flu, SJW, or SG for 8 weeks by oral gavage, whereas rats of the HC and CUMS group were administered with equal volumes of sterile water. The antidepressants or sterile water were administered between 9 and 11 a.m. before the CUMS procedures over the course of 8 weeks. After the last administration, the blood samples were immediately collected from the orbital veins and then performed behavioral tests. One day after the completion of the SPT (Day 85) and the OFT (Day 86), fecal samples were collected and all rats were then sacrificed to harvest adrenal samples after abdominal anesthesia with 10% chloral hydrate (0.3 ml/100 g).

### Preparation of SG and *Hypericum perforatum* L. Extract

*Hypericum perforatum* L. extract (Lot: K187264) was purchased from Xi’an Kailai Biological Engineering Co., Ltd. (Xi’an, China). It was collected in the city of Xi’an, Shaanxi Province, China (33^°^57^′^N, 107^°^37^′^E). SG (Lot: 200912) was provided by Sichuan Jishengtang Pharmaceutical Co., Ltd. (Sichuan Province, China). *Eleutherococcus senticosus* (Rupr. and Maxim.) Maxim. and *Hypericum perforatum* L. in SG were collected in the city of Pengzhou, Sichuan Province, China (30^°^54^′^N, 104^°^10^′^E).

### Phytochemical Characterization of SG by LC-MS/MS

The HPLC-mass spectrometry analysis (LC-MS/MS) was used to identify the six compounds (Hyperoside, Quercetin, Pseudohypericin, Hypericin, Eleutheroside E, and Isofraxidin) in SG samples (Xiao et al., 2018). LC-MS/MS analysis was performed on a Shimadzu LC-30AD HPLC system (Shimadzu Corp., Kyoto, Japan) and an AB Sciex Qtrap^®^ 5500 MS/MS (AB Sciex, Framingham, MA, United States) using a Welch Ultimate XB-C_18_ analytical column (2.1 mm × 100 mm, 4.6 μm, Shanghai, China), maintained at 40°C. LC conditions: flow rate 0.355 ml/min; injection volume 5 μL; mobile phase A: 0.1% formic acid-water solution (containing 5 mmol L^−1^ ammonium acetate); mobile phase B: acetonitrile (containing 0.1% formic acid); gradient elution procedure: 0–5 min, 10%B; 5–8 min, 10%-75%B; 8–11 min, 75%-97%B; 11–18 min, 97%B; 18–20 min, 97%-10%B; 20–25 min, 10%B; 25.10 min stop. MS conditions: The mass spectrometer was operated under the multiple reaction monitoring (MRM) protocol in positive ion mode (ESI+) and negative ion mode (ESI-) using an electrospray atmospheric pressure ionization source. The drying and nebulizing gas was nitrogen. The MRM transitions and the related optimized declustering potential (DP), entrance potential (EP), collision energy (CE), and collision cell exit potential (CXP) for different analytes are shown in Supplementary Table S1. MRM data was obtained and the chromatograms were integrated by the Analyst 1.6.1 software.

All reference substances were purchased from Shanghai Zhenzhun Biology Company (Shanghai, China). We accurately weighed the reference substances of hyperoside (4.20 mg, Lot No: ZZS19032507), quercetin (5.30 mg, Lot No: ZZS18122403), pseudohypericin (4.00 mg, Lot No: ZZS18103001), hypericin (4.60 mg, Lot No: ZZS18103008), eleutheroside E (4.00 mg, Lot No: ZZS19050508) and isofraxidin (4.70 mg, Lot No: ZZS18041705) by XS205DU electronic balance (Mettler Toledo, Greifensee, Switzerland). Then we placed them in a 10 ml volumetric flask, dissolved and diluted them to the mark with methanol (Eleutheroside E with 50% methanol), and obtained a standard stock solution with a concentration of 200 μg/ml. The standard stock solutions were stored with proper labeling at −20°C. A standard mixture of six main components with a concentration of 1000 ng/ml was prepared by mixing a stock solution of each and diluting it to 10 ml.

The quantity of the capsule contents equivalent to 8.70 mg of SG was transferred into a 10 ml calibrated flask and dissolved in 9 ml of 50% methanol. The capsule powder was ultra-sonicated (500 W, 53 kHz) with 50% methanol for 30 min, with 1 ml 50% methanol added to 10 ml and filtrated through a 0.22 μm membrane filter. Finally, 50% methanol was used to dilute the filtrate to 500 ng/ml with serial dilutions.

### Chronic Unpredictable Mild Stress

The CUMS procedure combined previous literature methods with those established in our research group (Zhai et al., 2015). Briefly, the CUMS group (n = 60) was exposed to various unpredictable stressors and was randomly given one or two kinds of stimulation every day. These stressors included: 1) light/dark (12/12 h) cycle reversing; 2) 2 h behavior restriction; 3) forced swimming at 40°C for 5 min; 4) electric shock for 10 s; 5) exposure at 0°C for 10 min; 6) water deprivation for 24 h; 7) exposure to 40°C for 5 min; 8) food deprivation for 24 h; 9) tail squeezing (1 min). And the corresponding modeling stimulation was carried out according to Table 1. During the modeling process, the HC rats (n = 15) were housed in an adjacent room, having no contact with the model animals. We observed the weight and life status of the rats, and then evaluated the modeling results according to behavioral experiments after modeling.

**TABLE 1:** CUMS modeling.

*Stressors*	Experiment Date	Experiment Date	Experiment Date
*light/dark (12/12 h) cycle reversing*	1	16	25/28
*2 h behavior restriction*	2	15	24
*forced swimming at 40°C for 5 min*	3	12	21
*electric shock for 10s*	4	10	23
*exposure at 0°C for 10 min*	5	11	22
*water deprivation for 24 h*	6	14	20
*exposure to 40°C for 5 min*	7	13	19
*food deprivation for 24 h*	8	17	26
*tail squeezing (1 min)*	9	18	27

### Behavioral Testing

#### Sucrose preference test (SPT)

The anhedonia induced by the CUMS protocol was assessed by the SPT (Liu et al., 2017). In SPT, during the adaptation phase, rats were individually housed in cages and acclimatized to a 1% (w/v) sucrose solution before the test, with two bottles of sucrose solution being placed in each cage for 24 h. Following this period one bottle of 1% (w/v) sucrose solution was replaced by water for 24 h. After adaptation, the rats were deprived of water and food for 24 h, which was followed by ad libitum access to one bottle of a 1% (w/v) sucrose solution and another bottle of water for 1 h baseline test. The remaining volume of the consumed water and sucrose solution was measured and recorded. The sucrose preference (%) was calculated with the formula as follows: sucrose preference = sucrose consumption/(water consumption + sucrose consumption) × 100%.

#### Open field test (OFT)

As a model of anxiety and locomotor activity, the open field apparatus consisted of a square arena with black walls and a black base which was divided into 16 equal squares (Jiang et al., 2018). The rats were given approximately 30 s to acclimate before the start of the test started. Each rat was carefully placed in the center of an open arena and allowed to explore for 5 min. The total traveled distance and the number of squares passed by rats were recorded. After each trial, 75% ethyl alcohol was used to clean the open field apparatus, so to avoid the interference left behind by the other rats.

### Enzyme-Linked Immunosorbent Assay (ELISA) Analysis

Corticosterone and serotonin levels were measured by an ELISA kit (Abcam, ab108821 and Cloud-Clone Corp, CEA808Ge, respectively). After the last administration, blood samples were collected from the orbital veins (before depression and anxiety-like behavior tests were performed), and the plasma samples were separated by centrifugation at 3000×g for 10 min and stored at −80 °C until analysis.

### 16S rDNA Sequencing

Feces were collected before the rats were sacrificed, and immediately stored at -80 °C until analysis. A total of thirty fecal samples were randomly selected from all groups for intestinal flora analysis. The fecal samples were taken from six rats receiving CUMS treatment, six rats receiving Flu after CUMS, six rats receiving SJW intervention after CUMS, six rats receiving SG intervention after CUMS, and six rats under normal control. The total genomic DNA of collected fecal samples was extracted by the cetyltriethylammnonium bromide (CTAB) method, and the agarose gel electrophoresis method was applied to detect DNA integrity, purity, and concentration. Diluted genomic DNA served as a template, PCR was performed, and the PCR product was detected by 2% agarose gel electrophoresis, and the product was recovered using the GeneJET gel recovery kit provided by Thermo Scientific. The library was constructed by the Ion Plus Fragment Library Kit 48 rxns (ThermoFisher, Scientific, Waltham, MA, United States). The constructed library was quantified by Qubit, received testing, and was sequenced by Ion S5TMXL (ThermoFisher, Scientific, Waltham, MA, United States).

### Data Analysis Process and Method

After the raw data was processed, the sequences were clustered into operational taxonomic units (OTU) that contained 97% similarity. According to the OTU clustering results, species annotation was made for the representative sequence of each OTU, and to obtain the corresponding species information and species-based abundance distribution. We applied the Uparse algorithm (Uparse v7.0.1001, http://www.drive5.com/uparse/) to cluster the sequences into OTUs with 97% identity (Edgar, 2013). Then we utilized the Mothur method (Wang et al., 2007), LVA132 SSUrRNA database (http://www.arb-silva.de/) (Quast et al., 2013), and MUSCLE (Version 3.8.31, http://www.drive5.com/muscle/) (Edgar, 2004) to perform species annotations on OTU sequences and analyze the relative abundance of intestinal flora.

#### Alpha diversity

Alpha diversity was used to analyze the microbial community diversity of the within-community (Li et al., 2013). Alpha diversity index (Shannon, Simpson, chao1, ACE, goods coverage, PD whole tree), rarefaction curve, and group rank abundance curve were served to assess the differences in species richness and diversity of microbial communities in each sample. Qiime software (Version 1.9.1) was applied to calculate the alpha diversity index; R software (Version 2.15.3) was used to draw the dilution curve and rank abundance curve. Difference analysis between groups of alpha diversity index was processed using R software. The alpha diversity index differences were analyzed by parametric and non-parametric tests. Given there were only two groups, the *t*-test and Wilcox tests were applied. For more than two groups, Tukey’s test and the Wilcox test of the Agricola package were used.

#### Beta diversity

Beta diversity represented a comparative analysis of the microbial community constitutions with different samples. The unweighted UniFrac distance was calculated according to phylogenetic relationships of OTUs (Lozupone and Knight, 2005; Lozupone et al., 2011). The weighted UniFrac distance is on the basis of the OTU’s information abundance (Lozupone et al., 2007). The UniFrac distance was calculated using Qiime software (Version 1.9.1), and principal coordinate analysis (PcoA) was drawn by R software (Version 2.15.3). The PCoA analysis was performed by R software’s WGCNA, stats, and ggplot2 software packages. The *t*-test and Wilcox test were applied to analyze the differences between the two groups, while the Tukey test and Wilcox test of the Agricola package were applied to analyze more than two samples. Linear discriminant analysis effect size (LEfSe) software was used for the LEfSe analysis, which defaulted to a filter value of four for the linear discriminant analysis (LDA) score. Metastats analysis was carried out under various classification levels by R software (Version 2.15.3) to obtain *p* value, and then applied Benjamini and Hochberg False Discovery Rate method to correct the *p* value, q value obtained.

#### Tax4Fun

Tax4Fun functional prediction was achieved by the nearest neighbor method based on the minimum 16S rRNA sequence similarity. BLASTN algorithm, SILVA SSU Ref NR database (BLAST bitscore > 1500), KEGG database, UProC, and PAUDA methods were applied to make Tax4Fun function prediction.

The experimental data involving more than two groups and/or time points were analyzed by one-way ANOVA followed by Tukey’s multiple comparisons tests using GraphPad Prism 7.0 software (GraphPad Software, San Diego, CA, United States). All data were presented as mean ± standard deviation. *p* < 0.05 was considered statistically significant.

## Results

### LC-MS/MS Analysis of SG

**FIGURE 2:**
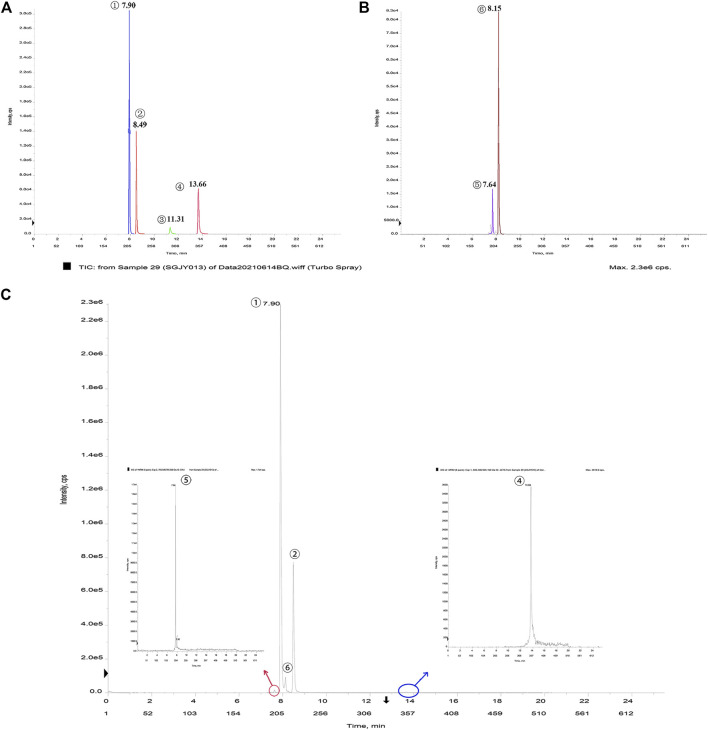
**(A) (B)**LC-MS/MS analysis of the main chemical compounds found in SG. Retention times:①Hyperoside, 7.90 min ②Quercetin, 8.49 min ③Pseudohypericin, 11.31 min ④Hypericin, 13.66 min ⑤Eleutheroside E, 7.64 min ⑥Isofraxidin, 8.15 min **(C)**LC-MS/MS analysis of SG (500 ng/ml).

The main chemical compositions of the six compounds (Hypericin, Hyperoside. Pseudohypericin, Quercetin, Eleutheroside E, Isofraxidin) in SG were detected by LC-MS/MS, see Figure 2A,B. LC-MS/MS analysis of SG (500 ng/ml) is shown in Figure 2C.

### SG Has a Significant Therapeutic Effect on CUMS Model Rats

**FIGURE 3:**
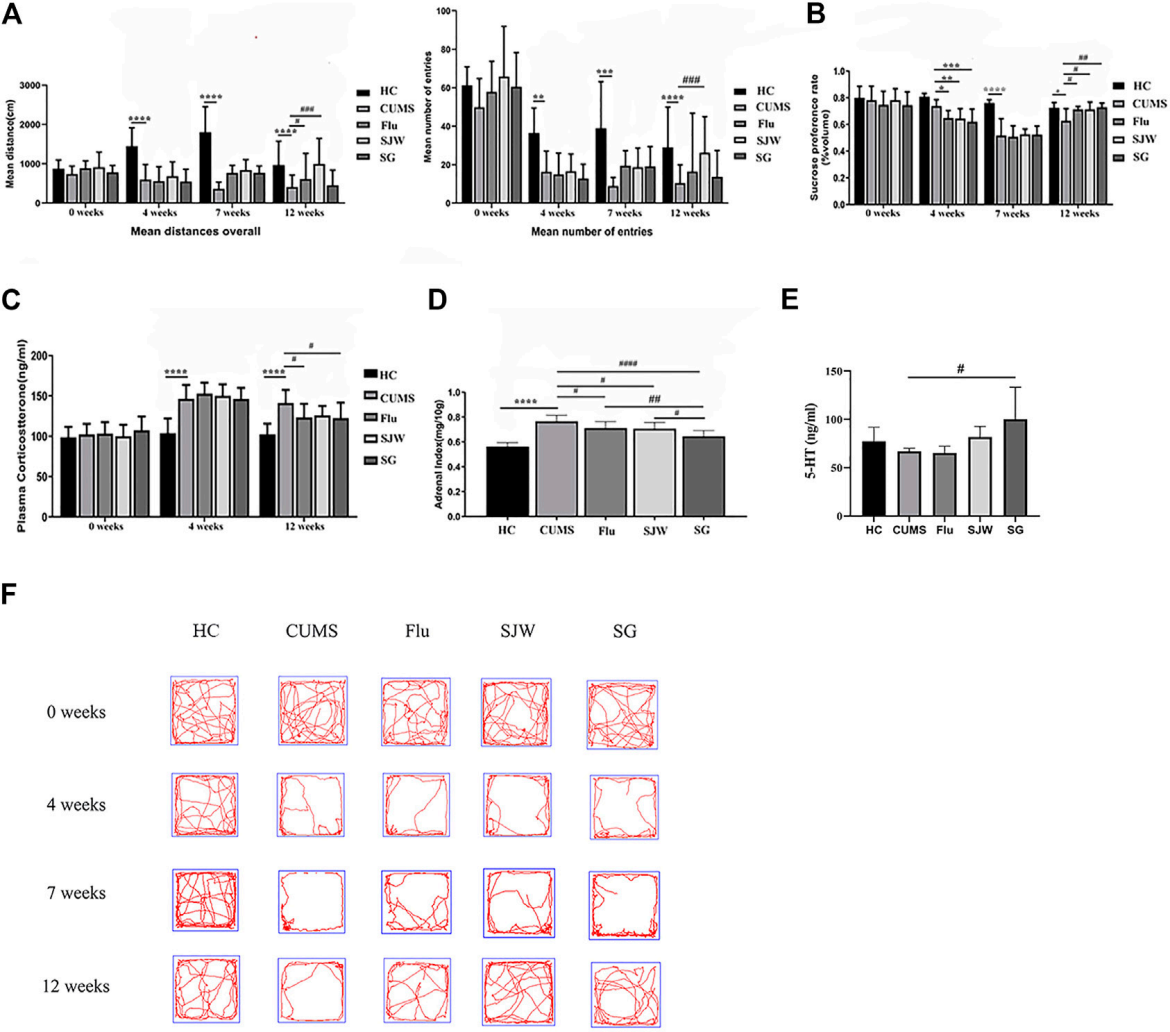
**(A)** OFT shows the mean distance and number of squares crossed (mean number of entries). **(B)** SPT shows the sucrose preference index. **(C)** the plasma corticosterone levels. **(D)** the adrenal index. **(E)** plasma 5-HT levels (n = 6 per). **(F)** The action roadmap of each group in the OFT. Statistical significance of the CUMS group versus the HC group was represented as ^****^ *p* < 0.0001; ^***^ *p* < 0.001; ^**^ *p* < 0.01; ^*^ *p* < 0.05; Statistical significance of the three administration groups versus the untreated CUMS group was represented as ^####^ *p* < 0.0001; ^###^ *p* < 0.001; ^##^ *p* < 0.01; ^#^ *p* < 0.05.

**FIGURE 4:**
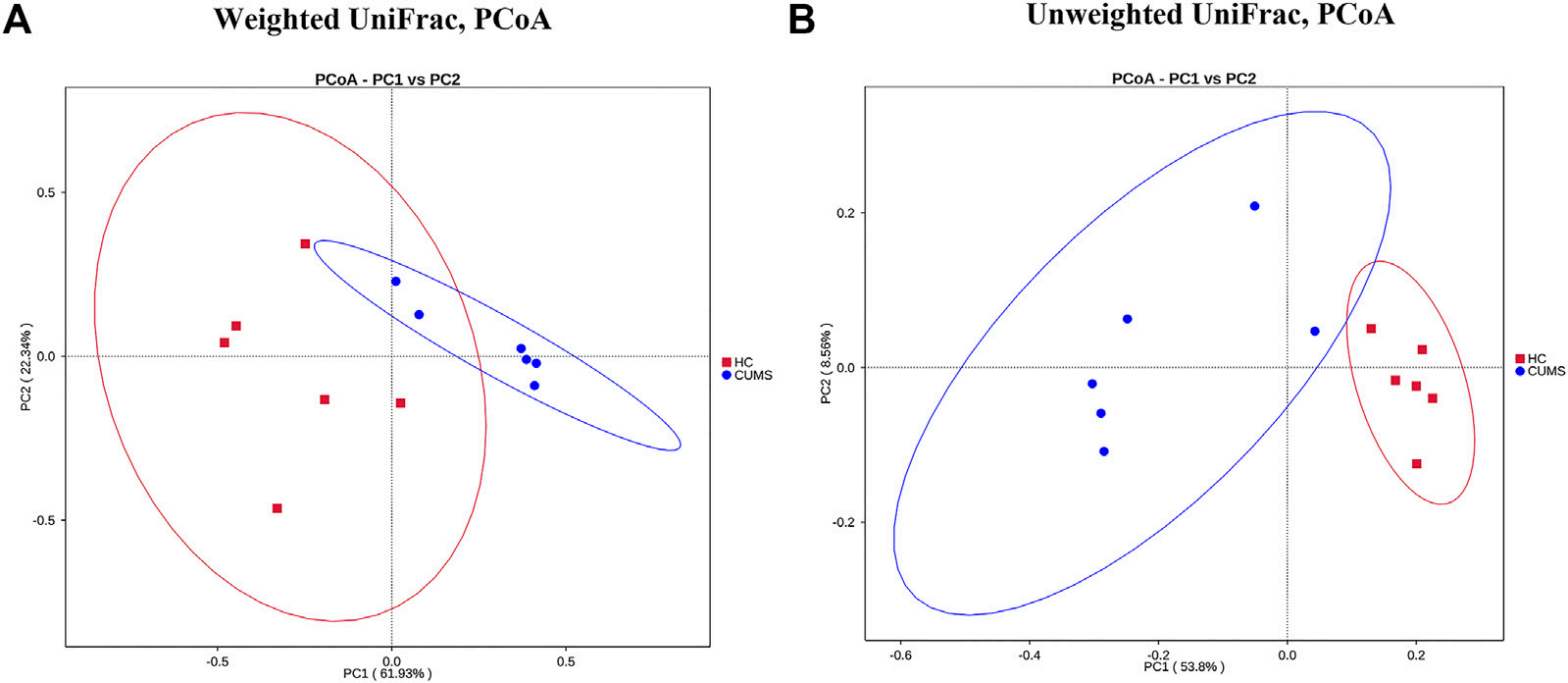
PCoA plots of bacterial beta-diversity on the basis of Weighted UniFrac distance **(A)** and the Unweighted UniFrac distance **(B)**. HC (red circles) vs CUMS (blue circles) groups.

After 4 weeks of model establishment, the SPT and OFT results showed that the CUMS model group was significantly different from the HC group (Figure 3A,B). The corticosterone concentration of rats in the CUMS model increased significantly (Figure 3C); the adrenal index also significantly increased (Figure 3D). These results were consistent with previous studies, indicating that the CUMS procedure successfully induced depression (Rubin et al., 1996; Herzog et al., 2009; Chakraborty et al., 2020; Gu et al., 2020; Stoneham et al., 2021; Xu et al., 2022). At the end of 12 weeks, we investigated the serotonin level in plasma samples and observed the trend of a decreased concentration of 5-HT in the CUMS rats. Treatment with SG could significantly increase the concentration of 5-HT in plasma (*p* < 0.05) (Figure 3E). After 8 weeks of treatment with Flu, SG, and SJW extract, behavioral tests demonstrated that some of the depressive-like behaviors of rats induced by CUMS were rescued. For example, by the end of the study, all three treatment groups showed a recovery in the sucrose preference index, indicating a decrease in anhedonia. By the final week of treatment, the Flu and SJW treatment improved the OFT results, with an increase in total distance and number of squares crossed compared to the untreated group. But such a phenomenon was not seen in the SG group. The action roadmap of each group in the OFT was shown in Figure 3F. After the administration of SG, both the final corticosterone levels in the plasma and the adrenal index decreased significantly, compared to the untreated groups. The above results indicated that after 8 weeks of treatment, SG could have antidepressant effects similar to Flu and SJW on rats with CUMS-induced depression, although the effect was not seen in OFT. Notably, the PCoA analysis performed on the basis of the Weighted UniFrac distance and the Unweighted UniFrac distance showed that the intestinal flora of HC was clustered and separated from rats in CUMS model, which further proved the successful establishment of CUMS model (*p* < 0.05) (Figure 4; Supplementary Table S2).

### Administration of SG Changes the Alpha and Beta Diversity of Intestinal Flora

**FIGURE 5:**
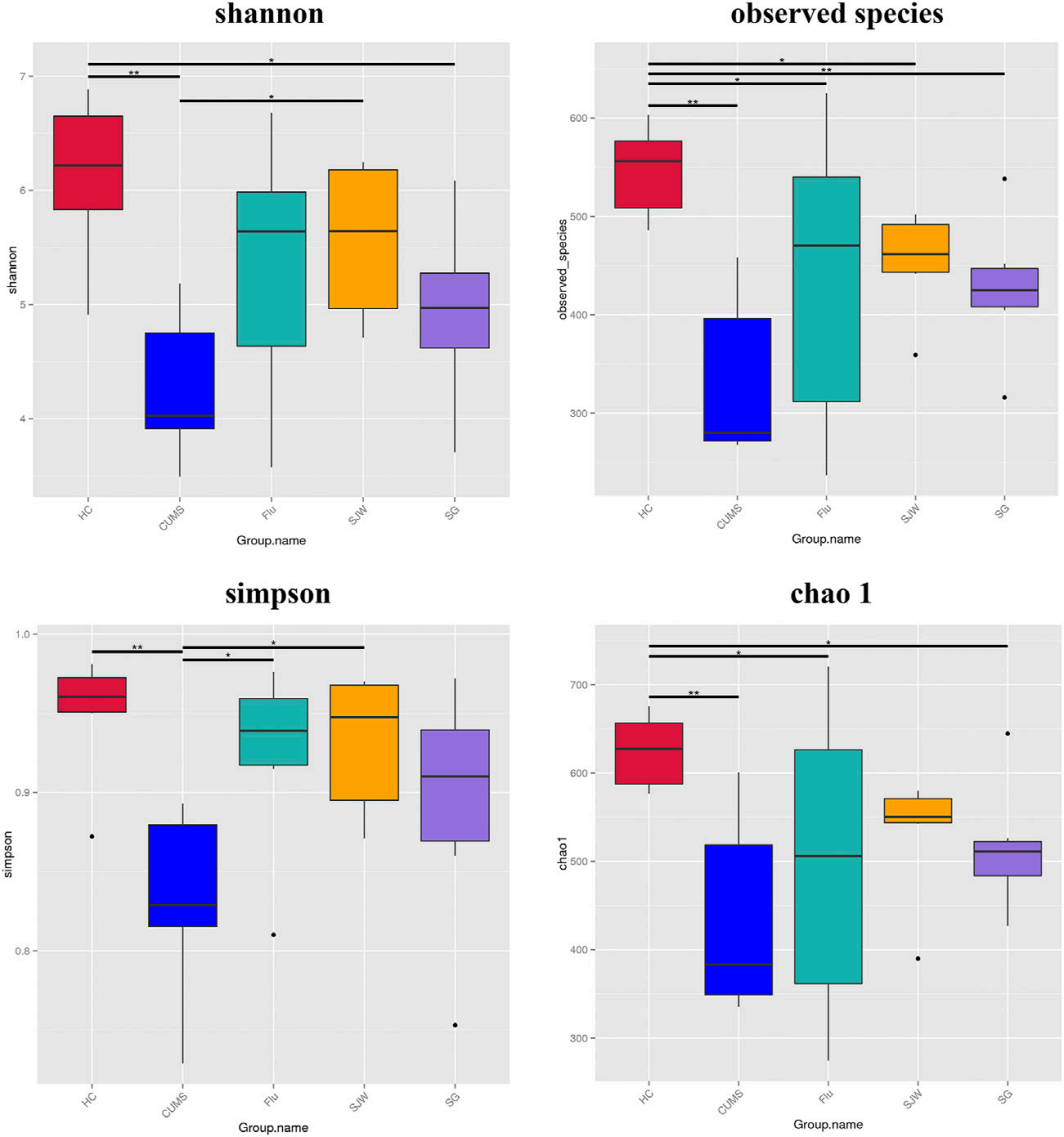
Alpha diversity analysis index, including the chao1 index, Shannon index, Simpson index, and observed species (^****^ *p* < 0.0001; ^***^ *p* < 0.001; ^**^ *p* < 0.01; ^*^ *p* < 0.05).

**FIGURE 6:**
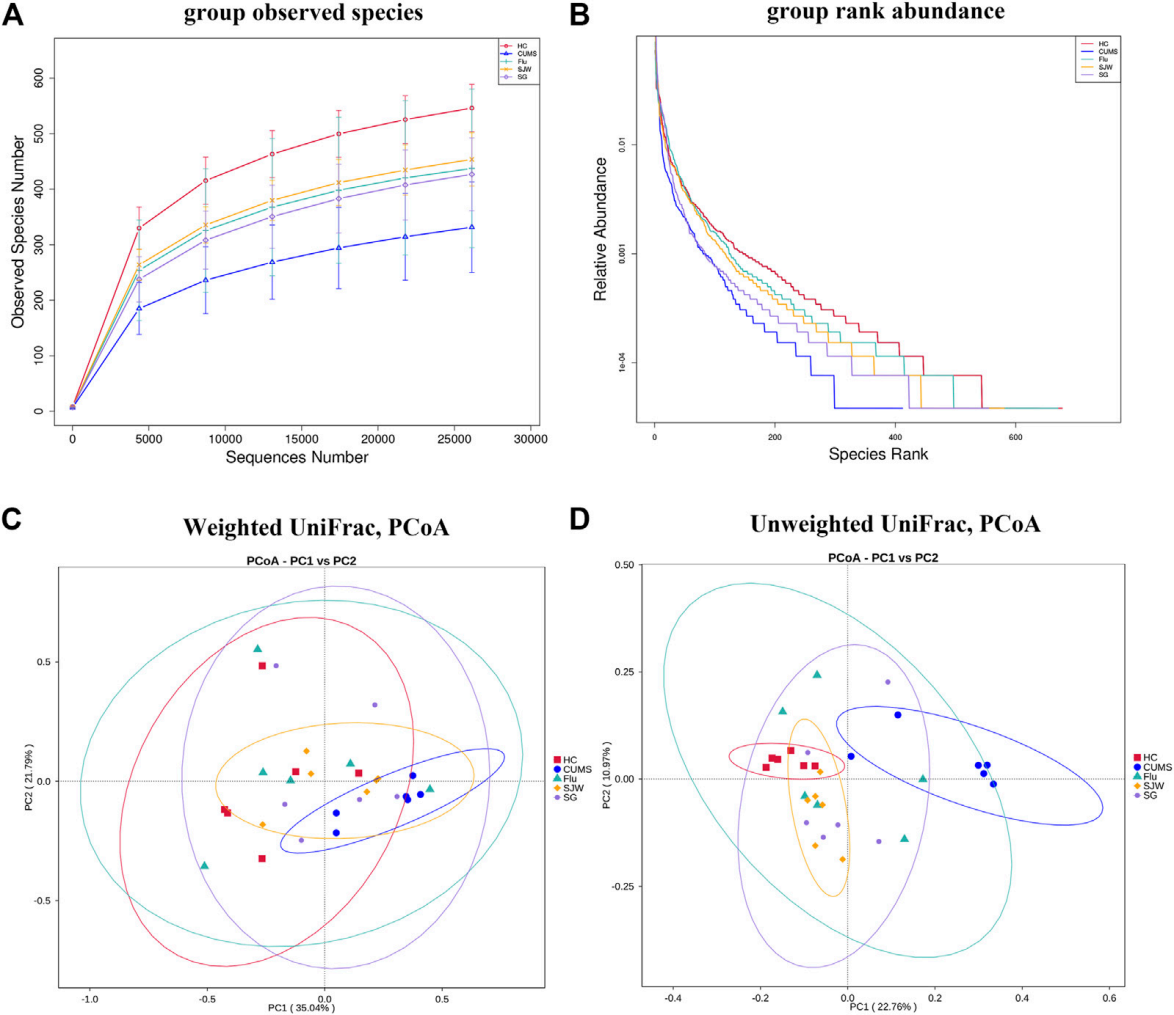
**(A)** Rarefaction curve. The abscissa is the number of sampling sequences and the ordinate is the number of observed species. When the curve tends to be flat, it indicates that the amount of sequencing data is gradually reasonable, and indirectly reflects the abundance of species in the sample. **(B)** Group rank abundance curve. In the horizontal direction, the larger the curve width is, the higher the species abundance is; in the vertical direction, the smoother the curve is, the more even the species distribution is. **(C)** PCoA plots of bacterial beta-diversity on the basis of Weighted UniFrac distances. **(D)** PCoA plots of bacterial beta-diversity on the basis of Unweighted UniFrac distances.

Statistics on the alpha diversity analysis index (Shannon, Simpson, chao1, ACE, goods coverage, PD whole tree) of different samples under the 97% consistency threshold, revealed that the sample coverage of each group exceeds 0.995, indicating that the accuracy of sequencing is reliable and the sequencing depth has basically covered all species in the sample (Supplementary Table S3). The chao1 index, Shannon index, Simpson index, and observed species were selected to analyze diversity, with each of these showing a significant decrease in diversity in the CUMS group (*p* < 0.01) (Figure 5). Shannon index, an alpha diversity measure of both microbial richness and evenness, was enhanced after SJW treatment (*p* < 0.05; Figure 5; Supplementary Table S4). Observed diversity, a measurement of OTU number, increased after treatment. Although there were no statistically significant differences (*p* > 0.05; Figure 5; Supplementary Table S4). Simpson index, also served as an alpha-diversity measurement of both microbial richness and evenness, enhanced following Flu and SJW treatment (*p* < 0.05; Figure 5; Supplementary Table S4). Chao 1, an index used to reflect species richness, increased in three treatment groups, but there were no statistically significant differences (*p* > 0.05; Figure 5; Supplementary Table S4). A comparison between the CUMS and administration groups demonstrated that, while there was no significant difference observed in bacterial diversity after treatment with SG, the richness and uniformity of the flora increased and the sequencing results were reliable (Figure 6A,B).

Calculating the difference in beta diversity allowed us to compare the composition of microbial community in different groups. We performed PCoA analysis based on the Weighted UniFrac distance and the Unweighted UniFrac distance and selected the main coordinate combination with the largest contribution rate for map display. The axes of the PCoA of the Weighted UniFrac distances explained 35.04 and 21.79% of the variation between the communities (Figure 6C), while the PCoA using the Unweighted UniFrac distances had axes explaining 22.76 and 10.97% of the variation between communities (Figure 6D). PCoA on Unweighted UniFrac distances showed a clearer separation than Weighted UniFrac distances. After administration, there was a significant separation between the HC group and CUMS group, the CUMS group, and Flu group, as well as the CUMS group and SG group, indicating the rich diversity of flora composition (*p* < 0.05; Supplementary Table S5).

### Administration of SG changes the relative abundance of intestinal flora

**FIGURE 7:**
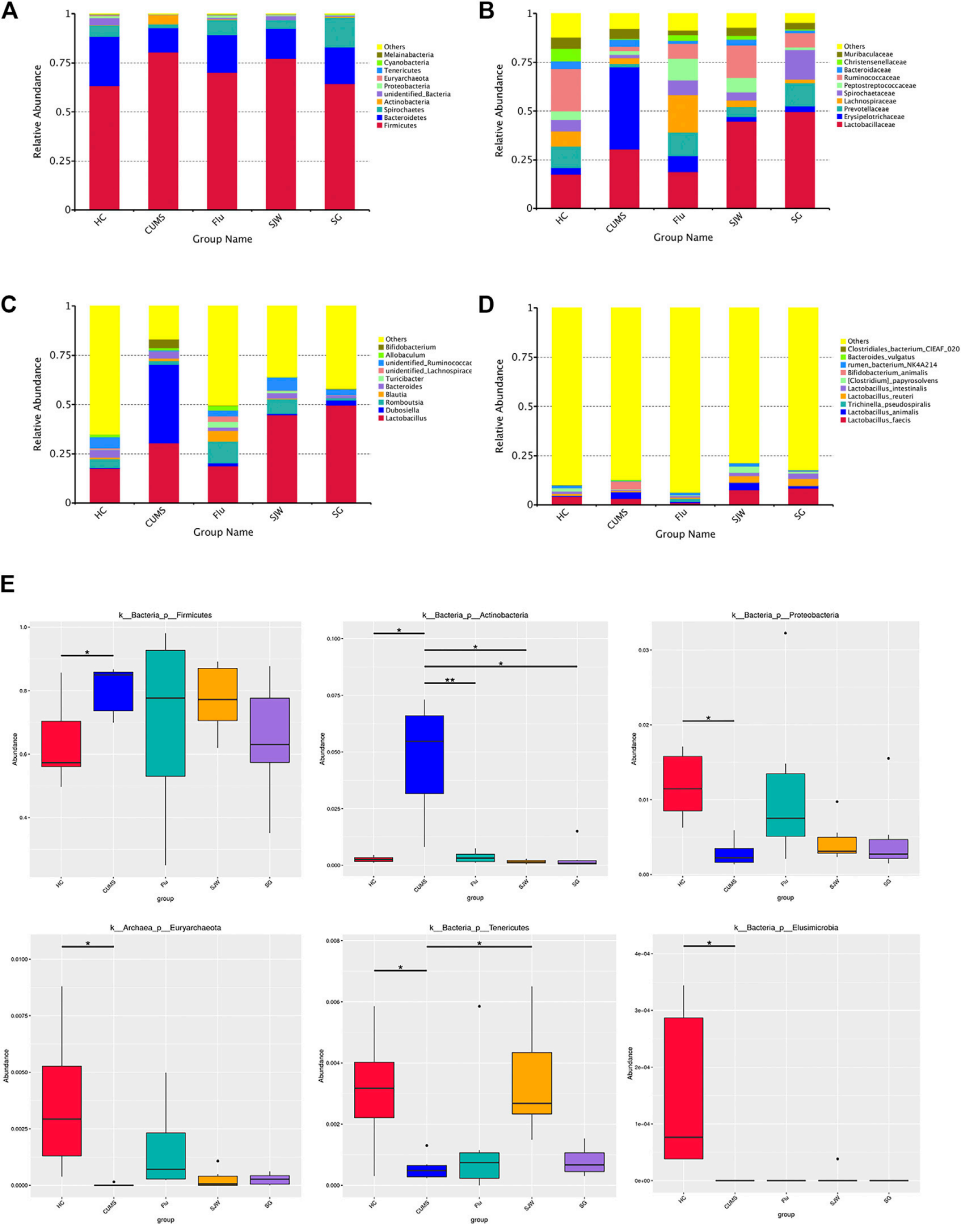
Fecal microbiota changes following antidepressant treatments at the phylum, family, genus, and species levels. Community bar-plot analysis showed the relative abundance of sequences at the phylum **(A)**, family **(B)**, genus **(C)**, and species **(D)** levels. **(E)**The MetaStat method was used to test the species abundance data between groups, correct the *p*-value, and then obtain the q-value. The statistics of the relative bacterial phyla abundances in HC, CUMS, Flu, SJW, and SG groups. Significant differences were indicated: ^*^q < 0.05, ^**^q < 0.01, ^***^q < 0.001. (k, kingdom level; p, phylum level).

According to the species annotation results of OTUs, the top 10 species with the highest abundance of each sample were selected (phylum, family, genus, and species), and generated a columnar cumulative map of species relative abundance to visualize the samples at different classification levels, species with the higher relative abundance and proportion. (Figure 7A–D).

At the phylum level, *Firmicutes*, *Bacteroidetes*, *Proteobacteria, Cyanobacteria,* and *Tenericutes* were the most abundant taxonomic groups. Lower ratio of *Firmicutes/Bacteroidetes* was observed in groups administered antidepressants (Supplementary Table S6). The relative abundances of *Firmicutes* and *Actinobacteria* in the CUMS model rats significantly increased compared with the HC group (*q* < 0.05; Figure 7E), and the relative abundance of *Proteobacteria*, *Euryarchaeota*, *Tenericutes,* and *Elusimicrobi* decreased (*q* < 0.05; Figure 7E). After treatment with Flu, SG, and SJW, the relative abundance of *Actinobacteria* significantly decreased (*q* < 0.05; Figure 7E). Interestingly, the relative abundance of *Tenericutes* only significantly increased in the SJW group (*q* < 0.05; Figure 7E).

At the family level, the relative abundance of Erysipelotrichaceae, Bifidobacteriaceae, and Atopobiaceae significantly increased in the CUMS-model rats compared with the HC group (*q* < 0.05; Supplementary Figure S7F), while the relative abundance of Ruminococcaceae, Christensenellaceae, and Lachnospiraceae decreased (*q* < 0.05; Supplementary Figure S7F); after administration with treatment, some of the flora changes were rescued. We observed that the level of Erysipelotrichaceae, Bifidobacteriaceae*,* and Atopobiaceae returned to the HC group in all treatment groups (*q* < 0.05; Supplementary Figure S7F). SJW administration significantly increased the relative abundance of Ruminococcaceae (*q* < 0.01), however this change was not observed in the Flu group and SG group (*q* > 0.05; Supplementary Figure S7F).

At the genus level, we observed that *Dubosiella* and *Bifidobacterium* increased significantly in the CUMS group (*q* < 0.01), and the three administration groups resulted in a return to the HC group levels after treatment (*q* < 0.01; Supplementary Figure S7G). Interestingly, the relative genus abundance that was unable to be identified in the Ruminococcaceae family increased significantly after SG and SJW treatment (*q* < 0.01), however Flu had no significant effect. The changes of these unidentified Ruminococcaceae may be related to the mechanism explaining the benefit of SG in treating depressive models (Supplementary Figure S7G).

We continued to observe the changes of intestinal flora at the species level and found that the levels of *Lactobacillus-animalis* and *Bifidobacterium-animalis* significantly increased in the CUMS model (*q* < 0.01), with *Eubacterium-ventriosum* significantly reduced (*q* < 0.01; Supplementary Figure S7H).

LEfSe analysis can realize the comparison between multiple groups to find the species with significant differences (i.e., biomarkers) in abundance between groups and assess the effect on them. First of all, we performed LEfSe analysis to realize the comparison between the HC group, CUMS group, and Flu group to find the species with significant differences in abundance. LDA indicated that four species in the Flu group had LDA scores >4.0, which was composed of *Clostridia* in class level, *Clostridiales* in order level, unidentified Lachnospiraceae in genus level, and *Turicibacter* in genus level (Supplementary Figure S7I). To find biomarkers for differentiating SJW from SG, LDA of LEfSe was used to determine the taxa that explain the differences between the SJW group and the SG group. The results revealed that five species in the SJW group had LDA scores >4.0, which was composed of unidentified Ruminococcaceae in genus level, *Romboutsia* in genus level, Peptostreptococcaceae in family level, *Clostridium-papyrosolvens* in species level, and *Lactobacillus-animalis* in species level. Meanwhile, Lactobacillaceae in family level, *Lactobacillus* in genus level, *Lactobacillales* in order level, *Bacilli* in class level, and *Lactobacillus-reuteri* in species level had LDA scores >4.0 in the SG group (Supplementary Figure S7J).

### Predicting the Gene Function of Intestinal Flora Using the Tax4Fun Tool

**FIGURE 8:**
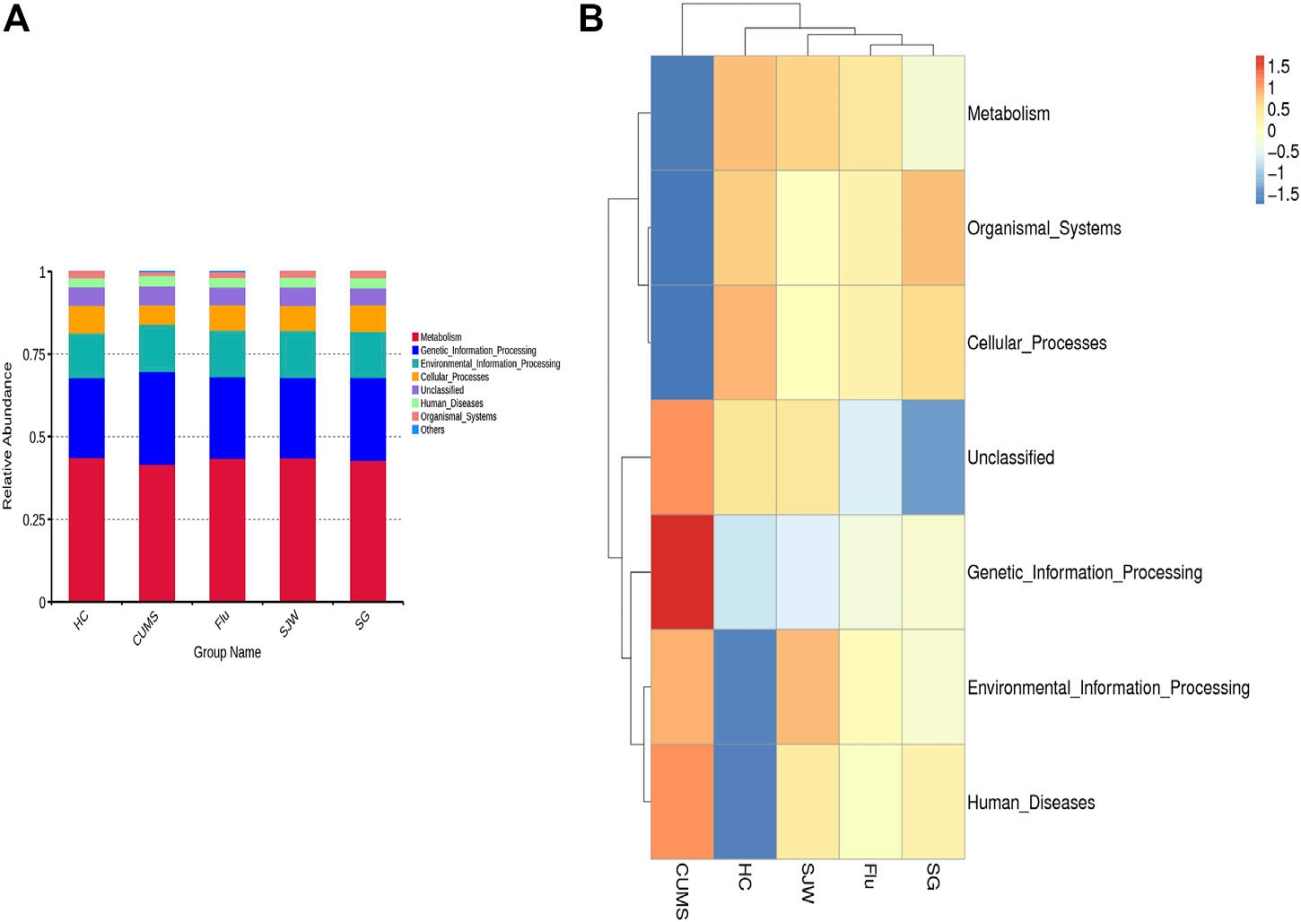
**(A)** Histogram of functional annotation relative abundance between the five groups on level 1. **(B)** Functional annotation clustering heat map using Tax4Fun on level 1.

To further understand the functional information related to changes in the intestinal flora, we used the Tax4Fun function to annotate the clustering heat map on level 1, 2, and 3. According to the database annotation results, we selected the functional information ranking the top 10 in the maximum abundance of each group, and generated the cumulative histogram of relative abundances of functions, to visually view the functions and their proportions with the high relative abundance of each group. At level 1, the functional genes of the five groups were mainly involved in metabolism, genetic information processing, environmental information processing, and cellular processes (Figure 8A). The top 35 abundance functions and their abundance information in each sample were selected to draw a heat map, and clustering was carried out from the functional difference level, according to the functional annotation and abundance information of the samples in the database. At level 1, compared with the CUMS group, administration of Flu, SG and SJW significantly increased the “metabolite”, “organic system” and “cellular processes” pathways and were consistent with the enriched functions in the HC group. But the changed functions of “Environmental Information Processing”, “Genetic Information Processing”, and “Human Diseases” were significantly reduced, which were inconsistent with the enriched functions in the HC group (Figure 8B). At level 2, the functional genes of the five groups were mainly involved in membrane transport, carbohydrate metabolism, replication and repair, translation, amino acid metabolism, nucleotide metabolism, energy metabolism, glycan biosynthesis and metabolism, signal transduction, and metabolism of cofactors and vitamins (Supplementary Figure S8C). We found that the SG group, SJW group, and HC group had the same enriched functions compared to the CUMS group, such as “immune system”, “amino acid metabolism”, “carbohydrate metabolism”, “signal transduction”, “metabolism of cofactors and vitamins”, “energy metabolism”, and “enzyme family pathways”. Surprisingly, “lipid metabolism” greatly increased in the SJW group; “xenobiotics biodegradation and metabolism” greatly increased in the SJW group, but decreased in the SG group (Supplementary Figure S8D). At level 3, the functional genes of the five groups were mainly involved in transporters, DNA repair and recombination proteins, transfer RNA biogenesis, purine metabolism, two component system, pyrimidine metabolism, ABC transporters, peptidases, ribosome, and amino acid related enzymes (Supplementary Figure S8E). The significantly different pathways seen between the various administration groups might indicate the unique targets of different drugs at level 3. Twenty-six pathways were consistently enriched in the three administration groups, including “ABC transporters”, “alanine, aspartate, and glutamate metabolism”, “amino acid related enzymes” “transporters”, “ribosome biogenesis”, “galactose metabolism”, and so on. The significantly different pathways between the SJW group and SG group are “mitochondrial biogenesis” and “quorum sensing”, as shown in Supplementary Figure S8F.

## Discussion

In this study, SG was shown to reduce the depressive symptoms of CUMS rats. Especially, we observed a significant decline of corticosterone levels and a significant increase of 5-HT levels in the plasma of CUMS-model rats after treatment with SG. SG also significantly reduced the adrenal index, to an extent significantly more than Flu or SJW (*p* < 0.05). These data showed that SG can partially neutralize corticosterone overproduction and the HPA axis dysregulation, which may be one of the mechanisms explaining antidepressant effects.

We explored the role of intestinal flora in the treatment of depression, which has attracted much attention in recent years (Chudzik et al., 2021). Understanding the mechanisms where the gut microbiota communicates with the CNS would help in the treatment that targets at HPA axis. To date, there is little consensus in how the types of intestinal flora are altered between depressed patients and healthy volunteers (Liu Y. et al., 2016; Zheng et al., 2016; Chen JJ. et al., 2018; Barandouzi et al., 2020b). Previous studies do support that there is a significant difference between intestinal flora of depressed patients vs. healthy volunteers (Barandouzi et al., 2020a). In our study, compared with the HC group, the alpha diversity in the CUMS model was significantly reduced. In addition, the beta diversity analysis of the Weighted and Unweighted UniFrac indicators showed a different community composition of the intestinal flora between the CUMS-induced group and the HC group, demonstrating the potential effect of chronic unpredictable mild stress on the intestinal flora. Our data indicated that the ratio of *Firmicutes/Bacteroidetes* increased in rats in depressive model rats, consistent with previous studies (Song et al., 2019; Johnson et al., 2020; Zhang W. et al., 2021); The intestinal flora of the rats administered with Flu, SG, and SJW differed from the untreated CUMS group, and the ratio of *Firmicutes/Bacteroidetes* decreased: this suggests that a decrease in the ratio of *Firmicutes/Bacteroidetes* may be related to the relief of depressive symptoms, although any causal links require future work. A high abundance of *Actinobacteria* has been observed in patients with depression (Huang N. et al., 2019). Our results showed that treatment with Flu, SG, and SJW can significantly rescue the relative abundance of *Actinobacteria* in CUMS-induced rats. We found that the relative abundances of several bacterial phyla were altered in the CUMS-induced model group, including lower *Elusimicrobia*, *Euryarchaeota*, *Proteobacteria*, and *Tenericutes*. Our findings on levels of lower *Elusimicrobia*, *Euryarchaeota,* and *Tenericutes* are consistent with past studies (Yang et al., 2017; Huang HL. et al., 2019; Tillmann et al., 2019). Surprisingly, in SJW group alone, the abundance level of *Tenericutes* increased significantly. *Tenericutes* levels have been reported to increase in Parkinson’s mice that have been fasted to model diet treatment (Zhou et al., 2019). Our group firstly observe its diversification in the depressive model with SJW. Contradictory results in the levels of *Proteobacteria* have been previously reported, with some researchers reporting a high abundance in patients with major depressive disorder (Jiang et al., 2015), while another study found a lower abundance of this phylum in people with depression (Chen Z. et al., 2018), a result more consistent with our study. A mouse model of Alzheimer’s disease was characterized by an increase in Proteobacteria after 6 months, and an increase in this phylum has also been seen in patients with autism (De Angelis et al., 2013; Bäuerl et al., 2018).

At the family level, we found that the relative abundance of Erysipelotrichaceae increased in CUMS model. It has been reported that a higher abundance of the genus Erysipelotrichaceae *incertae sedis* is found in depressive people (Barandouzi et al., 2020a). The Erysipelotrichaceae family has also been reported to be very immunogenic (Palm et al., 2014). Erysipelotrichaceae, a family related to inflammation, is also abundant in Alzheimer’s disease (Bäuerl et al., 2018) and inflammatory bowel disease (Schaubeck et al., 2016). Our results demonstrated that Flu, SJW, and SG significantly reduced the level of Erysipelotrichaceae*,* which may affect the inflammatory pathways and result in a decrease in depression. A high abundance of Bifidobacteriaceae was observed in our depressive model and has also been found in people with depression (Barandouzi et al., 2020a). It has been reported that Bifidobacteriaceae may exert an antidepressant effect by increasing the concentration of monoamine neurotransmitter 5-HT in the brain regions (Savignac et al., 2016; Chen et al., 2021). Researchers have also found that a high abundance of this bacteria Bifidobacteriaceae was associated with high fecal gamma-aminobutyric acid (GABA) production (Altaib et al., 2021). A series of interesting studies have suggested that GABA is involved in the modulation of immune cell activity and is associated with different systemic and enteric inflammatory conditions (Auteri et al., 2015). At the same time, GABA is the most abundant inhibitory neurotransmitter in the CNS, and it is well known that a reduction of its function and levels in the brain are closely related to depression (Duman et al., 2019). It is clear that microbial GABA can pass from the gut to other organs, however the mechanisms by which this influence is communicated to the brain are still unclear (Mazzoli and Pessione, 2016). SJW and SG, by regulating Bifidobacteriaceae levels, may affect the level of central neurotransmitters. In addition, our results showed that the relative abundance of Lachnospiraceae, Ruminococcaceae, and Christensenellaceae significantly decreased in the depressive model group. A lower abundance of Christensenellaceae has previously been associated with affective disorders (Coello et al., 2021). Regarding depression, studies have observed positive correlations between brain-derived neurotrophic factors and the Lachnospiraceae family (Park et al., 2020). We did not find any significant change in Lachnospiraceae levels among the three treatment groups. Studies have found that the abundance of Ruminococcaceae is significantly lower in rats with depression than in normal rats, consistent with our findings (Zhu et al., 2019). The decreased population size of Ruminococcaceae is significantly correlated with increased intestinal permeability and is more likely to occur in the presence of intestinal inflammation (Leclercq et al., 2014). Among of three mentioned flora, we only observed a significant increase in the relative abundance of Ruminococcaceae after SJW treatment, which may indicate the role of SJW in SG. SG tended to increase the relative abundance of Ruminococcaceae*,* but we did not observe the statistically significant differences. The results of LEfSe analysis revealed that Peptostreptococcaceae was the bacteria with significant differences in abundance after treatment with SJW. Some evidence supports that Peptostreptococcaceae is the characteristic flora associated with depression. Researchers have found Peptostreptococcaceae is the intestinal-associated flora of patients with depression compared with healthy volunteers (Zheng et al., 2021). In the chronic variable stress-induced depression rat model, the relative abundances of Peptostreptococcaceae *incertae sedis* also significantly decreased (Yu et al., 2017). LEfSe analysis demonstrated that Lactobacillaceae significantly changed in SG group. Interestingly, high abundance in Lactobacillaceae was observed in people with depression (Barandouzi et al., 2020b). A study has found that Lactobacillaceae is the key bacteria in depressive-like behavior induced by lead exposure in rats (Chen et al., 2021).

At the genus level, we found that the relative abundance of *Dubosiella* and *Bifidobacterium* increased significantly in the depressive model rats and decreased significantly in the SJW group and SG group. Interestingly, researchers have found that *Dubosiella* in a model of colitis increases significantly and decreases significantly after antioxidant and anti-inflammatory treatment (Sun et al., 2019; Sheng et al., 2020). If SG increases the abundance of *Dubosiella*, of the family Erysipeotrichaceae, this may help reduce inflammation-caused damage. *Bifidobacterium* has a relatively high abundance among MDD patients, consistent with our findings (Chung et al., 2019). Therefore, *Bifidobacterium* of the family Bifidobacteriaceae may alter the level of neurotransmitters and explain SG’s ability to relieve depression. In our findings, SJW and SG can significantly reverse the level of decreased unidentified Ruminococcaceae, potentially explaining their antidepressant mechanisms, which may be related to intestinal permeability and intestinal inflammation. The results of LEfSe analysis revealed that *Turicibacter* was the flora with significant differences in abundance after treatment with Flu. High abundance of *Turicibacter* was found in people with depression (Barandouzi et al., 2020b). However, researchers have examined the effects of low-dose ketamine, known to induce antidepressant effects, on stool microbiome profile in adult Wistar male rats. They have found that ketamine strikingly amplified *Turicibacter* by 26 fold (Getachew et al., 2018). It is interesting that abundance of *Turicibacter* has been linked to intestinal cytokine expression, and its role in depression requires more research to elucidate (Liu W. et al., 2016; Szyszkowicz et al., 2017). LEfSe analysis showed that *Romboutsia* in genus level was the characteristic bacteria after treatment with SJW. Significant overgrowth of *Romboutsia* has been found among high trait anxiety populations, as well as depression patients (Zheng et al., 2021; Wang et al., 2022). *Lactobacillus* was observed as the species with significant differences in abundance after treatment with SG. *Lactobacillus* is known as beneficial bacteria. Combined with our previous MetaStat results, SG may stimulate the growth or activity of specific intestinal bacteria (genus *Lactobacillus* and *Bifidobacterium*), which are related to the health and welfare of the host (Paiva et al., 2020).

At the species level, SG and SJW significantly reversed the CUMS-model related increase in the relative abundance of *Bifidobacterium-animalis*. However, it was reported that probiotic intervention (given probiotic *Bifidobacterium animalis subsp*) can improve the state of anxiety in athletes under stressful situations and their performance under these conditions (Dong et al., 2020). While promising that probiotics may alleviate anxiety/depression, the underlying mechanisms need to be further explored. *Eubacterium-ventriosum* was found to significantly decrease in the CUMS model group. Both humans and rodents can transform corticosterone and its 5α-Ring A-reduced metabolites to 21-dehydroxylated products: 11β-OH-progesterone or 11β-OH-(allo)-5α-preganolones in the intestine by *Eubacterium-lentum* (Morris and Brem, 2019). This finding indicates that *Eubacterium* levels may help explain the relationship between corticosterone levels in plasma and intestinal flora. The LEfSe analysis showed that *Clostridium-papyrosolvens* and *Lactobacillus-animalis* in species level significantly changed flora with the treatment of SJW. *Clostridium-papyrosolvens,* also known as *Ruminiclostridium-papyrosolvens*, can produce a wide variety of carbohydrate-active enzymes to enhance cellulosic biomass degradation (Ren et al., 2019). A study suggested that *Lactobacillus-animalis* was involved in chronic stress-induced depression-like behaviors in mice (Ji et al., 2022). Some strains of *Lactobacillus-animalis*, such as *Lactobacillus-animalis* KCTC 3501 and *Lactobacillus-animalis* 30a-2, are currently used as probiotics in the food and animal breeding industry (Nam et al., 2011; Lin et al., 2020). *Lactobacillus-reuteri* in species level was the characteristic bacteria after treatment with SG. Treatment with *Lactobacillus-reuteri* could alleviate anxiety and depressive-like behavior in mice, and its antidepressive effects are possibly associated with improved gut microbiota and serotonin metabolism, as well as GABA-related mechanisms (Jang et al., 2019; Xie et al., 2020; Zhou et al., 2022).

We analyzed gene functional information related to changes in the intestinal flora after antidepressant administration and found that the changed pathways were basically consistent with the HC group at level 1. At level 2, we observed the enhancement of carbohydrate metabolism pathways in CUMS-model rats, which may indicate a higher energy requirement in depressed rats. We also found the enhancement of lipid metabolism, immune system, and amino acid metabolism in three antidepressant groups, showing close relation to the aforementioned flora. For example, *Bifidobacteria* has been reported to have effects on plasma lipid profiles in dyslipidemic patients (Guardamagna et al., 2014); *Lactobacilli* has been shown to play an important role in the regulation of immune and inflammatory pathways (Yang et al., 2020); And these two florae have also been reported to affect the ratio of kynurenine/tryptophan (Kazemi et al., 2019). The differences found in pathways are enriched between the untreated group, and the administration groups provide mechanistic insight into how the drugs work. Alanine, aspartate and glutamate metabolism were not significantly affected by SG and SJW treatment, but the Flu group had a decrease in all these pathways. Recent research reported that Flu and SJW can reduce glutamate and GABA release (Lazarevic et al., 2019). Glutamate is the metabolic precursor of GABA, so whether SG can regulate Glu/GABA balance may be worth considering in the future. Mitochondrial dysfunction is one of the biological mechanisms underlying the pathophysiology of MDD (Rappeneau et al., 2020). We found that the process of mitochondrial biogenesis up-regulated in the CUMS group and the SJW group brought it back towards the HC group levels, however we did not observe the trend in the SG group. It is worth noting that quorum sensing pathways strongly increased in the SG group. A past study has shown that the bacterial quorum-sensing molecule (AI-2) can be an important conduit of signals from the gut to the brain and an AI-2 inhibitor has antidepressant effects (Medina-Rodriguez et al., 2020). SG may participate in the process of quorum sensing to relieve depression. In general, these results support the effects of SG on the function of intestinal flora and provide clues into the potential mechanism for the relief of depression.

Our study showed that SG could ameliorate CUMS-induced depression, and the underlying mechanism may be associated with improvements in gut microbiota composition and changes in intestinal inflammation and glutamate metabolism. It is known that microorganisms and the brain communicate with each other through the immune system, tryptophan metabolism, vagus nerve, and enteric nervous system (Janssens et al., 2021). This communication involves a variety of microbial metabolites, such as pro-inflammatory factors, short-chain fatty acids, and neurotransmitters. Pro-inflammatory factors have been shown to affect the pathogenesis of Alzheimer’s disease (Zhang et al., 2020). Studies have reported that short-chain fatty acids, metabolites of intestinal microbes, can regulate the permeability of the blood-brain barrier, affect the expression of related proteins in the frontal cortex and hypothalamus, and participate in the regulation of microglial homeostasis (Generoso et al., 2020). Some researchers believe that glutamate, as a neuroactive molecule produced by intestinal microbes in the human body, participates in the important physiological functions of the CNS and enteric nervous system (Baj et al., 2019). Some microbial-derived intestinal metabolites may engage in the communications between intestinal microbes and the brain in depression, and help us better understand the microbiota-gut-brain axis. Whether the gut microbiota differences among CUMS, Flu, SJW, SG, and HC groups are a cause or the consequence of depression, they may have implications regarding specific diagnostic tests, and/or for treatment and prevention.

However, several limitations were present in our study. Some results of the intestinal flora were contrary to previous studies. These contradictions have also been reported in other studies and may be the result of a combination of many factors. We speculate that it is closely related to differences in the depressive model species and problems with small sample sizes. Also it would be more accurate to analyze microbial functioning when using taxonomic approaches to understanding the gut microbiome in depression. At the species level, due to the current limited detectable levels, we only observed a few changed characteristic bacteria. We believe that with the development of technology, more drug-related, important bacterial species will be detected. In addition, in this study, we observed different enriched pathways between the SG group and SJW group, suggesting possible mechanisms of *Eleutherococcus senticosus* (Rupr. and Maxim.) Maxim. in SG. Subsequent research can set the *Eleutherococcus senticosus* (Rupr. and Maxim.) Maxim. group to further explore its mechanisms. We ignored the effects of behavioral testing on the gut microbiota. The behavioral tests (OFT and SPT) in rats are also a potent psychophysiological stressor that may alter endocrine, neurochemical, immune function, and microbiota, but very little research has been done in this field (Pawlak et al., 2005; Gu et al., 2020; Xu et al., 2022).

In summary, SG was shown to alleviate depression-like behaviors and could partially rescue the function of the HPA axis. *Eubacterium* may be the key to explaining the relationship between the function of the HPA axis and intestinal flora. The 16S rDNA sequencing data indicated that SG influenced the abundances of *Dubosiella* of the family Erysipeotrichaceae and Ruminococcaceae*,* which are related to intestinal permeability and intestinal inflammation. In addition, SG lowered the abundance of Bifidobacteriaceae*,* which is thought to regulate GABA production and the level of neurotransmitters in the CNS. And our results supported that SG can regulate the level of neurotransmitters 5-HT in peripheral plasma and the role of Bifidobacteriaceae in it deserves further exploration. LEfSe analysis demonstrated that Lactobacillaceae (family level), *Lactobacillus* (genus level), *Lactobacillales* (order level), *Bacilli* (class level), and *Lactobacillus-reuteri* (species level) were biomarkers in the SG group samples. The antidepressive effects of *Lactobacillus-reuteri* also have been reported to associated with 5-HT metabolism, so its role in the antidepressant effect of SG is also worth exploring.

The present study demonstrates that SG can significantly alter the intestinal flora and the gut microbiome function in depressive model rats. Studying the changes in the structure and distribution of the intestinal flora, and then exploring the possible role of microbial-derived intestinal metabolites in depression will help us study the pathophysiology of depression in more detail. Our research provides new ideas for further research on Chinese antidepressants and provides potential targets to relieve depression.

## Data Availability

The original contributions presented in the study are publicly available. This data can be found here: figshare.com/s/8b993c2721cf36a7cafc.
