# Supplementary material for: Antidepressant Shugan Jieyu Capsule Alters Gut Microbiota and Intestinal Microbiome Function in Rats With Chronic Unpredictable Mild Stress -Induced Depression
Source: Front Pharmacol. 2022 Jun 13;13:828595. doi: 10.3389/fphar.2022.828595 (PMC9234866; doi:10.3389/fphar.2022.828595)
Supplement: Supplementary file 1 [file DataSheet1.docx]

## Supplementary data

**Table 1**

Retention time (RT), MRM transitions, declustering potential (DP), entrance potential (EP), collision energy (CE), and collision cell exit potential (CXP) of the analytes.

| Analytes | RT(min) | MRM transitions | DP(V) | EP(V) | CE(V) | CXP(V) |
| --- | --- | --- | --- | --- | --- | --- |
| Hyperoside | 7.90 | 463.2/300.0 | -92 | -4 | -41 | -15 |
| Quercetin | 8.49 | 301.2/151.1 | -66 | -11 | -32 | -23 |
| Pseudohypericin | 11.31 | 519.2/487.3 | -116 | -7 | -67 | -25 |
| Hypericin | 13.66 | 503.4/405.1 | -105 | -10 | -77 | -20 |
| Eleutheroside E | 7.64 | 765.5/765.5 | 213 | 9 | 7 | 21 |
| Isofraxidin | 8.15 | 223.1/162.2 | 69 | 9 | 35 | 26 |

**Table 2**

T-test and Wilcox tests of the bacterial fecal microbiota on the weighted and unweighted UniFrac distances between HC and CUMS groups (PCoA)

| Group-pair |  |  | P-Value |
| --- | --- | --- | --- |
| HC- CUMS | Weighted UniFrac  Unweighted UniFrac | T-test  Wilcox test  T-test  Wilcox test | 0.00158612090768686  0.00166521486418813  1.52719556103645e-06  3.86803502273631e-07 |

**Table 3** alpha diversity index

| group | Observed species | shannon | simpson | chao1 | ACE | Goods coverage | PD whole tree |
| --- | --- | --- | --- | --- | --- | --- | --- |
| HC  CUMS | 546  331 | 6.121  4.266 | 0.950  0.831 | 624.510  433.865 | 635.815  429.669 | 0.996  0.996 | 42.292  27.622 |
| Flu | 437 | 5.323 | 0.923 | 497.840 | 507.132 | 0.997 | 36.995 |
| SJW | 453 | 5.557 | 0.931 | 531.606 | 539.634 | 0.996 | 35.451 |
| SG | 426 | 4.935 | 0.892 | 515.784 | 520.394 | 0.996 | 35.731 |

**Table 4**

Wilcox tests of the alpha diversity index between HC, CUMS, Flu, SJW, and SG groups

| Group-pair | Shannon | P-Value  Observed species | Simpson | | Chao 1 |
| --- | --- | --- | --- | --- | --- |
| HC- CUMS | 0.0013 | 4e-04 | 0.002 | 0.0042 | |
| CUMS- Flu | 0.0599 | 0.0716 | 0.0183 | 0.3881 | |
| CUMS- SJW | 0.0173 | 0.0588 | 0.0141 | 0.212 | |
| CUMS- SG | 0.2731 | 0.2384 | 0.1152 | 0.471 | |

**Table 5**

TukeyHSD and Wilcox tests of the bacterial fecal microbiota on the weighted and unweighted UniFrac distances between HC, CUMS, Flu, SJW, and SG groups (PCoA)

| Group-pair |  |  | P-Value |
| --- | --- | --- | --- |
| HC- CUMS  CUMS- Flu  CUMS- SJW  CUMS- SG | Weighted UniFrac  Unweighted UniFrac  Weighted UniFrac  Unweighted UniFrac  Weighted UniFrac  Unweighted UniFrac  Weighted UniFrac  Unweighted UniFrac | TukeyHSD  Wilcox test  TukeyHSD  Wilcox test  TukeyHSD  Wilcox test  TukeyHSD  Wilcox test  TukeyHSD  Wilcox test  TukeyHSD  Wilcox test  TukeyHSD  Wilcox test  TukeyHSD  Wilcox test | 0.010704075806776  3e-04  0.00882337810655665  3e-04  4.35843614532416e-08  0  0.0311428901451719  0.0264  0.783024174257545  0.2406  0.583371990168247  0.1013  0.0110479024511406  8e-04  0.948342273074324  0.7347 |

**Table 6**

The relative abundances of *Firmicutes*, *Bacteroidetes*, and the ratio of *Firmicutes/Bacteroidetes* in all groups.

| group | Firmicutes | Bacteroidetes | Firmicutes/Bacteroidetes |
| --- | --- | --- | --- |
| HC | 0.633906 | 0.249879 | 2.536852 |
| CUMS | 0.804219 | 0.125223 | 6.422295 |
| Flu | 0.701576 | 0.191733 | 3.65913 |
| SJW | 0.773657 | 0.151877 | 5.093971 |
| SG | 0.644915 | 0.185683 | 3.473204 |

## Supplementary Figures


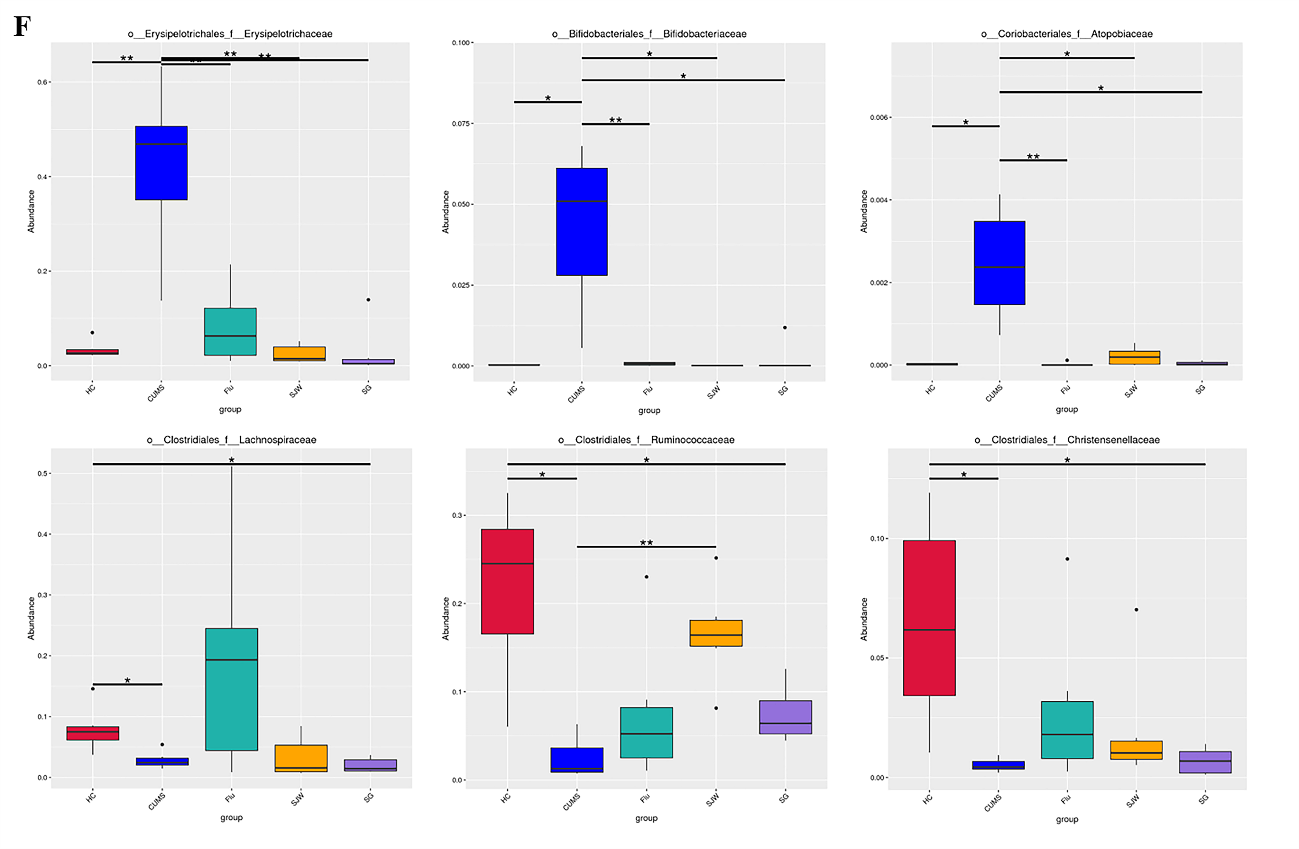


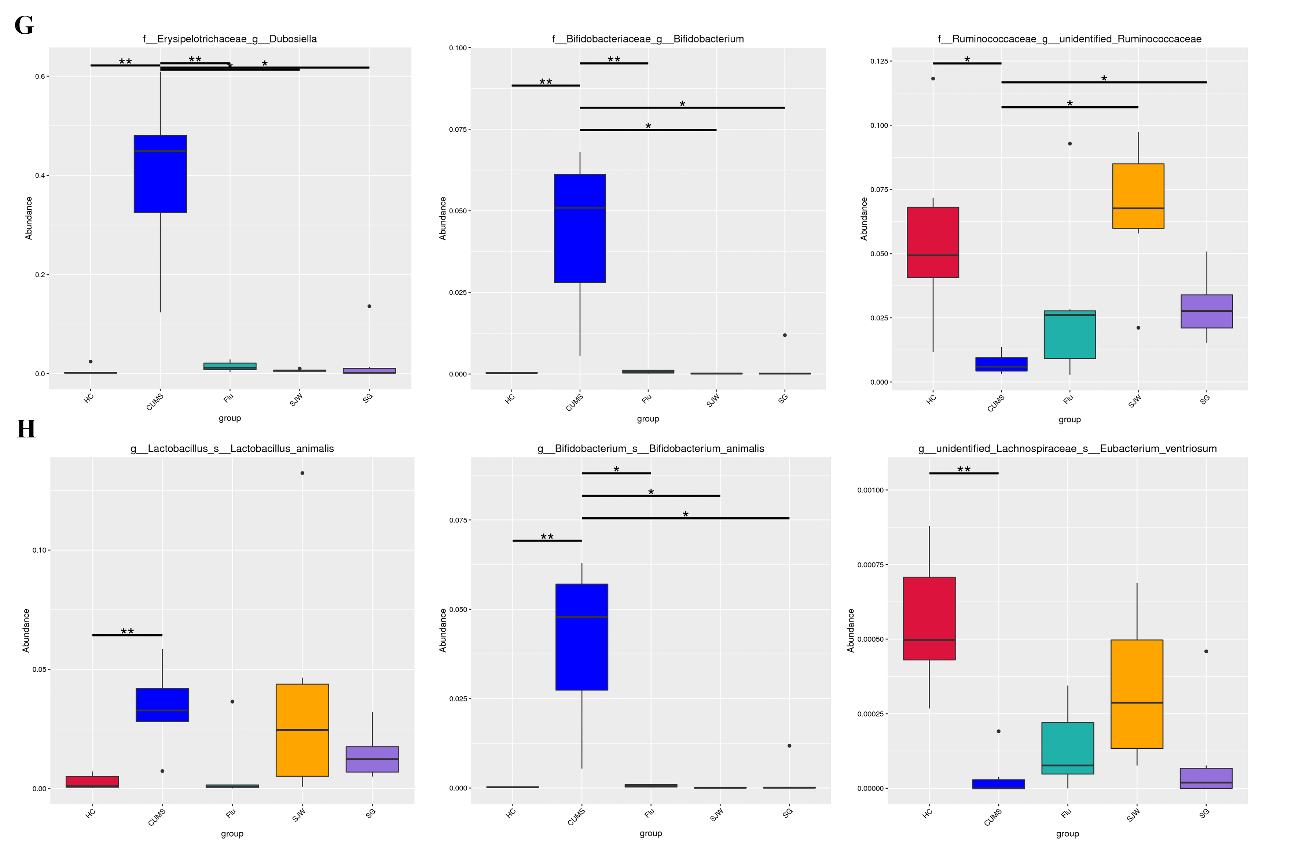


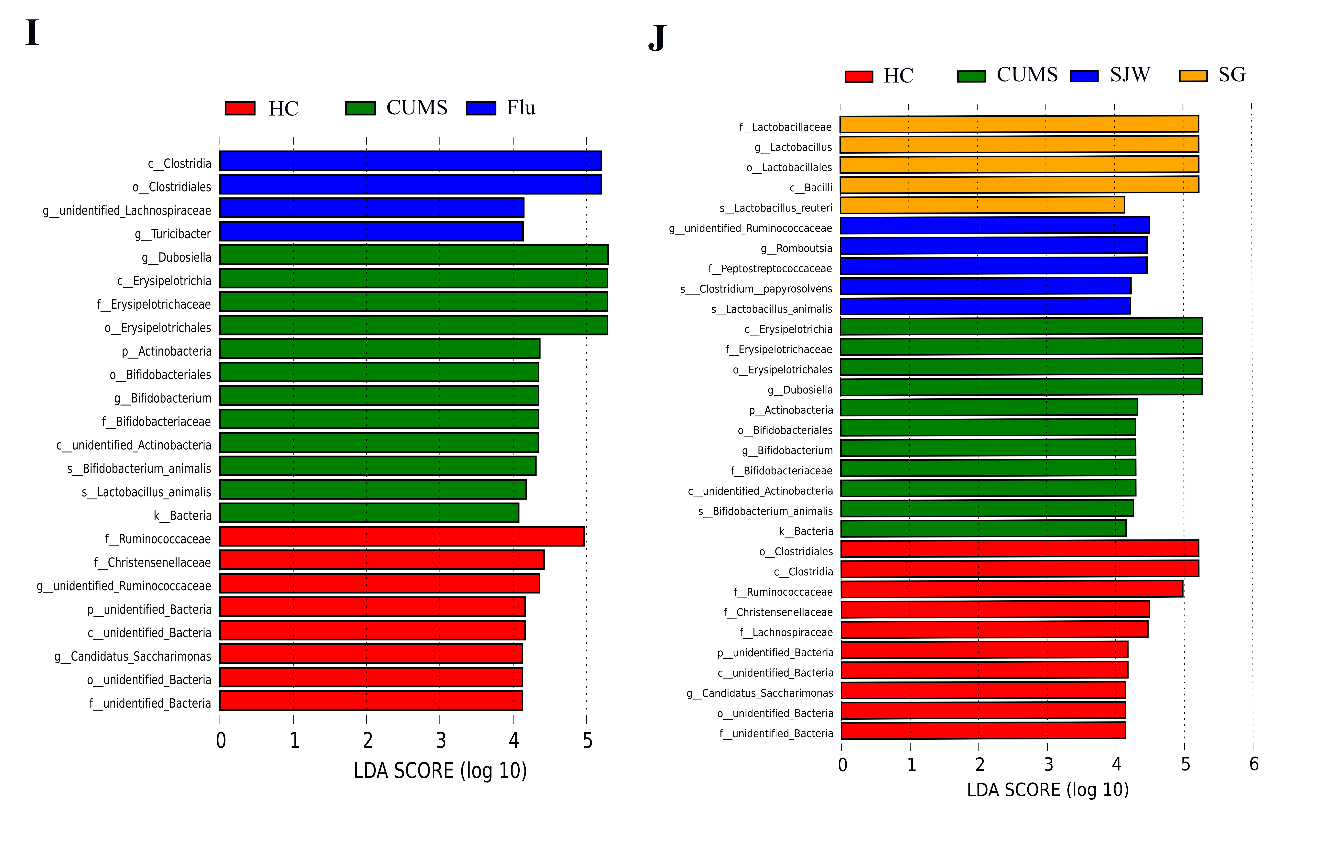


**Supplementary Figure 7.** The statistics of the relative bacterial family(F), genus(G), and species(H) abundances between groups using the MetaStat method. LDA score plot of microbial taxa with significant group differences. (I) LDA score plots of the HC, CUMS, and Flu groups. (J) LDA score plots of the HC, CUMS, SJW, and SG groups. Bacterial taxa with an LDA score of >4 were selected as the biomarker taxa (p, phylum level; c, class level; o, order level; f, family level; g, genus level; s, species level).


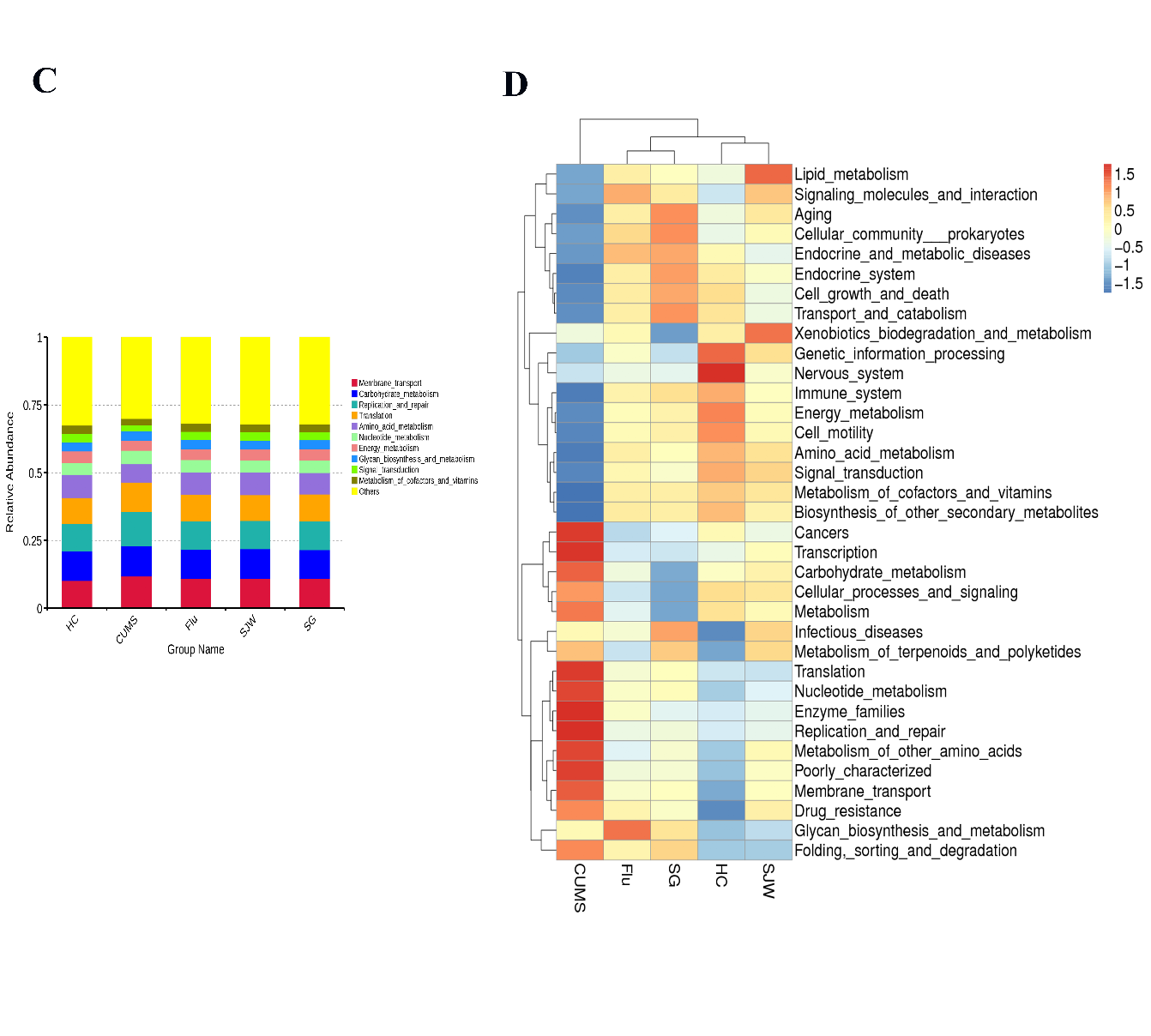


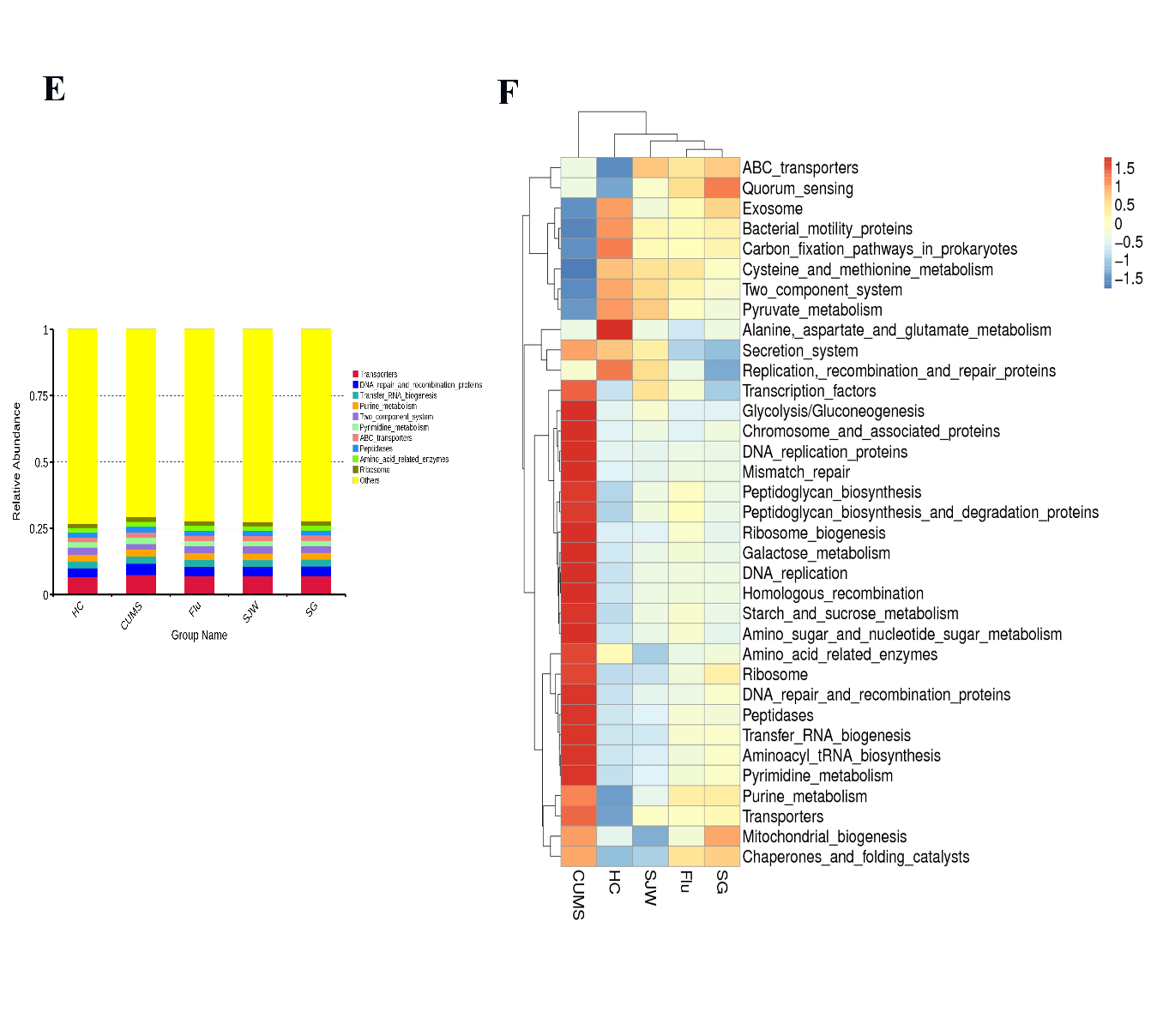


**Supplementary Figure 8.** Histogram of functional annotation relative abundance between the five groups and functional annotation clustering heat map using Tax4Fun on level 2(C) (D) and level 3(E) (F).
